# Entropy analysis and grey cluster analysis of multiple indexes of 5 kinds of genuine medicinal materials

**DOI:** 10.1038/s41598-022-10509-0

**Published:** 2022-04-22

**Authors:** Libing Zhou, Caiyun Jiang, Qingxia Lin

**Affiliations:** Guangxi Science & Technology Normal University, Laibin, 546199 Guangxi China

**Keywords:** Plant sciences, Chemistry

## Abstract

5 kinds of genuine medicinal materials, including Diding (Latin name: *Corydalis bungeana* Turcz), Purslane (Latin name: *Portulaca oleracea* L.), straw sandal board (Latin name: *Hoya carnosa* (L.f.) R. Br), June snow (Latin name: *Serissa japonica* (Thunb.) Thunb.), pine vine rattan (Latin name: *Lycopodiastrum casuarinoides* (Spring) Holub. [*Lycopodium casuarinoides* Spring]), were selected as the research objects. The combustion heat, thermo gravimetric parameters, and fat content, calcium content, trace element content, ash content of 5 kinds of genuine medicinal materials were measured. The combustion heat, differential thermal gravimetric analysis, fat content, calcium content, trace elements content, and ash content of 5 kinds of genuine medicinal materials were used to build a systematic multi-index evaluation system by gray pattern recognition and grey correlation coefficient cluster analysis, which can make up for the gaps in this area and provide scientific basis and research significance for the study of genuine medicinal materials quality. The results showed that the order of combustion heat of 5 kinds of genuine medicinal materials, including Diding, Purslane, straw sandal board, June snow, pine vine rattan, was Diding > June snow > straw sandal board > Purslane > pine vine rattan, the order of fat content (%) of 5 kinds of genuine medicinal materials was straw sandal board > Diding > pine vine rattan > June snow > Purslane, the order of calcium content (%) was pine vine rattan > June snow > Purslane > straw sandal board > Diding, the order of ash content was June snow > Purslane > straw sandal board > pine vine rattan > Diding. From the analysis of thermogravimetric analysis results and thermogravimetric combustion stability, the order of combustion stability of 5 kinds of genuine medicinal materials was June snow > pine Vine rattan > straw sandal board > Diding > Portulaca oleracea. The order of the content of 12 trace elements in 5 kinds of genuine medicinal materials, in terms of trace element content, June snow contains the highest trace elements in all samples. According to combustion heat, combustibility (combustion stability of genuine medicinal materials), fat, calcium, ash, trace element content, the comprehensive evaluation results of multi-index analysis constructed by gray correlation degree, gray correlation coefficient factor analysis, and gray hierarchical cluster analysis showed that the comprehensive evaluation multi-index order of 5 genuine medicinal materials, including Diding, Purslane, straw sandal board, June snow and pine vine rattan, was June snow > straw sandal board > Diding > Purslane > pine vine rattan. Therefore, the comprehensive evaluation results of the quality of genuine medicinal materials selected in this study were June snow the best, followed by straw sandal board. This research has important theoretical and practical significance for the multi-index measurement and comprehensive evaluation of genuine medicinal materials, and can provide scientific basis and research significance for the research of multi-index quality control of genuine medicinal material.

## Introduction

Guangxi, with its unique geographical conditions and humid and warm climate, is very suitable for the growth of Yao medicine and the planting of traditional Chinese medicine, making Guangxi one of the producing areas of traditional Chinese medicine in China, known as “genuine medicine”. In Laibin, Guangxi, the mountains and rivers are beautiful and the climate is pleasant, which is very suitable for the cultivation of Chinese medicinal materials^1,2^. Diding has the functions of clearing heat and dampness, detoxifying and reducing swelling; curing boils, carbuncle, scrofula, jaundice, dysentery, diarrhea, red eyes, throat numbness, snake bites and other effects^3^. Straw sandal board has the effects of clearing heat, dispersing blood stasis, promoting water and removing dampness^4,5^. Purslane has clearing heat and detoxification, cooling blood to stop bleeding, and stop dysentery. June snow has the effects of dispersing wind, relieving exterior, clearing heat and dampness, relaxing muscles and activating collaterals^6,7^. Pine vine rattan has the function of relaxing tendons and activating blood circulation, and has the effects of treating rheumatic joint pain, traumatic injury, muscle and bone pain and so on.

Modern research has proved that the curative effect of traditional Chinese medicine is not only related to organic ingredients, but also closely related to the types and contents of inorganic elements^8^. In addition to being essential elements for the body to participate in and regulate metabolism, inorganic elements also have a strong ability to form complexes, it is easy to form coordination bonds with ligands containing nitrogen, oxygen and sulfur in organisms, coordinate the balance of substances in the body, and form effective ingredients. Therefore, the synergistic effect of inorganic elements on the efficacy of the drug cannot be ignored.

The thermal analysis is simple, rapid, without pre-preparation, reproducible and easy analysis. In recent years, thermal analysis has a good application prospect in the research of traditional Chinese medicine. By reference to relevant domestic and foreign literatures, this essay summarizes the advance in studies on thermal analysis in terms of identification of traditional Chinese medicine and Chinese medicine preparation, to provide a reference for the application and extension of thermal analysis in the field of traditional Chinese medicine^9^.

Traditional Chinese medicine is a complex system of mixed chemical components, relying on a variety of chemical components to play a comprehensive therapeutic effect. The compatibility and therapeutic effects of traditional Chinese medicines are closely related to the energy effects of traditional Chinese medicines. When the medicine enters the body under the action of metabolic enzymes in the body, it will inevitably be accompanied by the release of energy of Chinese medicine chemicals. From a holistic perspective, it is necessary to conduct a macroscopic and comprehensive analysis of traditional Chinese medicine to control the quality of traditional Chinese medicine^10^.

The effective ingredients of traditional Chinese medicines and their preparations are complex, and it is difficult for a single-component quality control method to comprehensively and effectively control the overall quality of the product. Multi-index component quantitative control mode has been gradually applied to quality control of traditional Chinese medicine^11^.

The above studies are basically based on the quality evaluation or cluster analysis of a single index or active ingredient or some organic components on genuine medicinal materials. There are few studies on grey pattern recognition and cluster analysis from the multi-indexes such as trace elements, combustion heat, thermogravimetric analysis, fat, calcium, and energy of genuine medicinal materials. However, there are no relevant reports on the study of genuine medicinal materials stability by thermogravimetric method. Therefore, according to the thermogravimetric parameters, the entropy method is used to construct the combustibility (combustion stability of genuine medicinal materials) of Diding, Purslane, straw sandal board, June snow, pine vine rattan in different regions of China. The research on the combustion heat and thermogravimetric analysis of genuine medicinal materials has important theoretical and practical significance;the combustion heat, differential thermal gravimetric analysis, fat content, calcium content, trace elements content, and ash content of 5 kinds of genuine medicinal materials were used to build a systematic multi-index evaluation system by gray pattern recognition and grey correlation coefficient cluster analysis, which can make up for the gaps in this area and provide scientific basis and research significance for the study of genuine medicinal materials quality.

5 kinds of genuine medicinal materials, including Diding, Purslane, straw sandal board, June snow, pine vine rattan, were selected as the research objects. The combustion heat^12–17^ was measured by oxygen bomb calorimeter, the combustion stability^18–20^ of genuine medicinal materials was analyzed by thermogravimetric analysis, fat content was measured by fat analyzer^21–24^. According to the combustion heat, thermogravimetric parameters, and fat content, calcium content, trace element content, ash content, the multi-index comprehensive evaluation system^25,26^ of five kinds of genuine medicinal materials was established, and the entropy method and cluster analysis method were used to evaluate multiple indicators. The quality of genuine medicinal materials was evaluated by stoichiometric method from the aspect of multiple indicators, which provided a strong scientific basis for the large-scale development of genuine medicinal materials resources and the research of genuine medicinal materials classification.

## Methods

5 kinds of materials, including wild Diding (Latin name: *Corydalis bungeana* Turcz), Purslane (Latin name: *Portulaca oleracea* L.), straw sandal board (Latin name: *Hoya carnosa* (L.f.) R. Br), June snow (Latin name: *Serissa japonica* (Thunb.) Thunb.), pine vine rattan (Latin name: *Lycopodiastrum casuarinoides* (Spring) Holub. [*Lycopodium casuarinoides* Spring]), were purchased from Laibin traditional Chinese medicine market in June 2020, in Guangxi, China, and were selected as the research objects and the analysis samples, all samples were sieved through 40 mesh pharmacopoeia. Repeat the test multiple times for each sample to reduce errors. The Diding plant was identified as dry whole grass of Papaveraceae (Fig. S1), and *Portulaca oleracea* plant was identified as dry whole herb of *Portulaca oleracea* L. (Fig. S2), and straw sandal board was the dry leaf of dicotyledonous plant medicine bulbus of Asclepiadaceae (Fig. S3), and June snow was a dry whole herb of *Serissa serissoides* (DC.) Druce of Rubiaceae (Fig. S4), and pine vine rattan was the dry stem of a plant of the *Convolvulaceae fangchiaceae* (Fig. S5). The above materials GKS20190600010, GKS20190600011, GKS20190600012, GKS20190600013 and GKS20190600014 were respectively identified from the source, eye observation, hand touch, nose smell, mouth taste, comparison, etc. by Prof. Caiyun Jiang of Guangxi Science & Technology Normal University and were stored in Guangxi Science & Technology Normal University. Figures S1–S5 are in Supplementary Materials.

### Determination method of genuine medicinal material combustion heat

In this experiment, bomb calorimeter was used to determine the heat values under constant volume. The data processing formula is:

$$ {\text{mQv}} = {\text{W}}_{{{\text{cal}}}} \Delta {\text{T}} - {\text{Q}}_{{\text{ignition wire}}} \Delta {\text{m}} - {\text{ Q}}_{{{\text{capsule}}}} \,{\text{m}}_{{{\text{capsule}}}} . $$

In the formula: m, Qv, ∆T, W_cal_, Q_ignition wire_, ∆m respectively are the quality of the sample to be tested, the constant volume combustion heat, the temperature change before and after combustion, the calorimeter water equivalent, and the combustion heat of the ignition wire (Q_ignition wire_ = 1400.8 J/g), the actual mass of the ignition wire participating in the combustion reaction^27^.

### Instruments and reagents

BH series combustion heat measurement experimental device, oxygen cylinder, oxygen meter, grinder, sheet press, ignition wire (nickel–chromium wire, Changsha Changxing Higher Education Instrument Equipment Co., Ltd.); electronic balance(model FA2004, Shanghai Shunyu Hengping Scientific Instrument Co., Ltd.), benzoic acid (AR, Tianjin KERMEL Chemical Reagent Co., Ltd.), medicinal capsules.

### Thermogravimetric analysis method^28–30^

Thermogravimetric analysis is a technology to measure the relationship between the mass change of samples and temperature or time under a programmed temperature and a certain atmosphere. The temperature values on the curves are often used to compare the thermal stability of samples, which is the data basis for evaluating the combustibility of combustibles.

An appropriate amount of 2–10 mg samples were placed into an alumina crucible with a heating rate of 10 °C min^−1^, a reference compound of ɑ-Al_2_O_3_, N_2_ atmosphere (flow rate of 100 mL min^−1^), and a temperature range of 30–600 °C. TG, DTG and DTA analysis were performed at the same time. In order to reduce the experimental error, the experiment was repeated for each sample^31^.

### Instruments and reagents

Thermogravimetric analyzer (Germany NETZSCH STA 2500), crucible.

### Fat determination method

Soxhlet extraction, the fat content was determined by gravimetric method, that is, after the solvent of the extraction sample was extracted, the fat of the measured substance was extracted from the sample, dried and weighed, and calculated^32–35^. W_fat_ = m_1_/m_2_ × 100%, m_1_ is the fat mass (g), m_2_ is the sample mass (g).

### Instruments and reagents

SE206 fat tester, analytical balance, filter paper, 100 mL beaker, drying oven, petroleum ether.

### Determination method of calcium content

Calcium and aminocarboxylate can form metal complexes quantitatively, and its stability is stronger than that of calcium and indicator^36–43^. In the appropriate pH range, EDTA was titrated with ammonia-carboxylate complexing agent. When the measurement was reached, EDTA captured calcium ions in the indicator complex, making the solution present the color of free indicator (end point). According to the amount of EDTA complexing agent, calcium content can be calculated.

### Instruments and reagents

Muffler furnace, electric furnace, crucible, electronic balance, alkaline titration tube, conical bottle (250 mL), capacity bottle (100 mL), 20% sodium hydroxide solution, 1:1 triethanolamine aqueous solution, calcium red indicator (1 g mixed with 99 g sodium chloride grinding), hydroxylamine hydrochloride (analytical purity), malachite green indicator, ethylenediamine tetraacetic acid disodium (EDTA, referred to as a certain mass plus distilled water, and then 25 mL 0.1 mol/mL calcium standard solution for titration).

### Ash determination method

The residual inorganic matter in genuine medicinal materials after burning is called ash^44–48^.

### Instruments and reagents

Muffle furnace, electric furnace, crucible, electronic balance.

### Determination method of trace elements in 5 kinds of genuine medicinal materials

#### Determination method of trace elements content

In this experiment, 5 kinds of genuine medicinal materials, including Diding, Purslane, straw sandal board, June snow, pine vine rattan, in Guangxi, China, are all medicinal materials produced^49–55^. After sampling the uniform sample has been crushed with a crushing grinder, accurately weigh the sample 0.3 g (accurate to 0.0001 g) to 50 mL digestion container. Add aqua regia 8 mL and hydrogen peroxide 3 mL to the container. After standing overnight, put it into microwave digestion until the sample is completely digested, and the acid is removed. After cooling, it is filtered and the volume is fixed to a 50 mL volumetric flask, and the instrument is tested. The measurement of 12 kinds of trace elements is in accordance with GB/T 30903-2014. At the same time, the reagent blank experiment was performed, and the number of collection points and the number of repetitions were both 6 times.

### Instruments and reagents

Agilent7700 ICP-MS (Agilent, USA), CEM MARS-6 microwave digestion instrument (CEM, USA), Milli-Q ultrapure water preparation system (Millipore, USA), AUY120 millionth electronic analytical balance (Shimadzu Company). Nitric acid, hydrogen peroxide (super pure, Guangzhou Chemical Reagent Co., Ltd.), self-made ultra-pure water.

### Multi-index comprehensive evaluation method

According to the combustion heat, thermogravimetric parameters, and fat content, calcium content, trace element content, ash content, the multi-index entropy method and grey correlation coefficient cluster analysis of five kinds of genuine medicinal materials were constructed in Guangxi, China.

### Statement

5 kinds of materials, including wild Diding (Latin name: *Corydalis bungeana* Turcz), Purslane (Latin name: *Portulaca oleracea* L.), straw sandal board (Latin name: *Hoya carnosa* (L.f.) R. Br), June snow (Latin name: *Serissa japonica* (Thunb.) Thunb.), pine vine rattan (Latin name: *Lycopodiastrum casuarinoides* (Spring) Holub. [*Lycopodium casuarinoides* Spring]), were purchased from Laibin traditional Chinese medicine market in June 2020, in Guangxi, China, and were selected as the research objects and the analysis samples, all samples were sieved through 40 mesh pharmacopoeia. Repeat the test multiple times for each sample to reduce errors. The Diding plant was identified as dry whole grass of Papaveraceae, and *Portulaca oleracea* plant was identified as dry whole herb of *Portulaca oleracea* L., and straw sandal board was the dry leaf of dicotyledonous plant medicine bulbus of Asclepiadaceae, and June snow was a dry whole herb of *Serissa serissoides* (DC.) Druce of Rubiaceae, and pine vine rattan was the dry stem of a plant of the *Convolvulaceae fangchiaceae*. The above specimens GKS20190600010, GKS20190600011, GKS20190600012, GKS20190600013 and GKS20190600014 were respectively identified by Prof. Caiyun Jiang of Guangxi Science & Technology Normal University, and were stored in Guangxi Science & Technology Normal University.

Studies comply with relevant institutional, national, and international guidelines and legislation, local and national regulations.

## Results

### Calculation of combustion heat of five kinds of genuine medicinal materials

**Figure 1:**
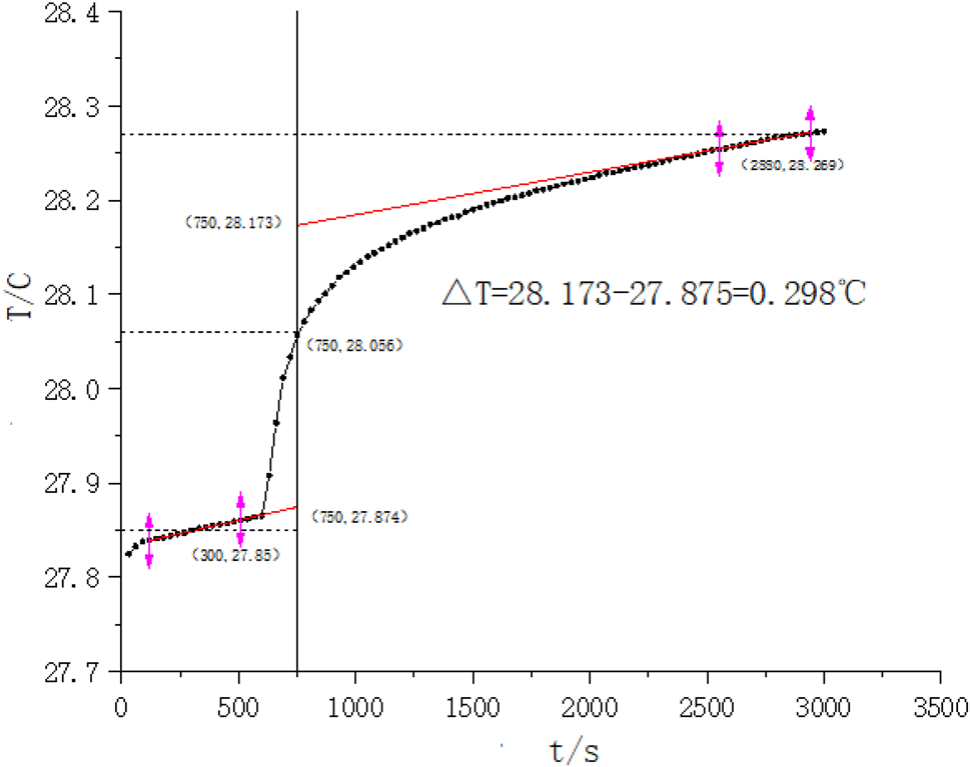
∆T curve of Reynolds temperature of Diding.

1. Sample name: the first group of experimental samples of Diding. According to the experimental data, ∆T Curve of Reynolds temperature of Diding is shown in Fig. 1. The experiment is repeated three times. Figure 1 shows ΔT Curve of Reynolds temperature. According to the calculation,
  $$ \Delta {\text{m}}_{\text{Diding}} = 0.0768\;{\text{g}},\;{\text{W}}_{\text{cal}} = 35054.24\;{\text{J}}/^\circ {\text{C}},\;Q_{\text{capsule}} = 60406.893\;{\text{J/g}},\;{\text{m}}_{\text{capsule}} = 0.0983\;g,\;\Delta {\text{T}} = 0.298\;^\circ {\text{C}}, $$
  ∆m_ignition wire_ = 0.0095 g, according to, ∆m_Diding_ Qv = W_cal_ ∆T−Q_ignition wire_ ∆m_ignition wire_−Q_capsule_ m_capsule_, Q_Diding_ = 58526.79 J/g. The average of Q_Diding_ is 56915.503 J/g.
2. In the same way, determine the combustion heat of Purslane, straw sandal board, June snow, pine vine rattan, and repeat the test for three times.The heat of combustion of 5 kinds of genuine medicinal materials, is shown in Table 1. According to Table 1, the order of combustion heat of 5 kinds of genuine medicinal materials, including Diding, Purslane, straw sandal board, June snow, pine vine rattan, was Diding > June snow > straw sandal board > Purslane > pine vine rattan. The combustion heat of the 5 kinds of genuine medicinal material test samples has ranged from 49,779.54 to 56,915.503 J/g, CV% < 3.65%.The combustion heat of Diding is 56,915.503 J/g, and the energy is the highest. The combustion heat of pine vine rattan is 49,779.548 J/g, and the energy is relatively small. Combustion heat is regarded as an important physical data to measure genuine medicinal material energy.

**Table 1:** Combustion heat of 5 genuine medicinal materials (n = 3).

Sample	Q_Vaverage_/(J g^−1^)	CV/%
Diding	56,915.503	0.0291
Purslane	52,124.475	0.0365
Straw sandal board	55,434.189	0.0313
June snow	55,607.597	0.0179
Pine vine rattan	49,779.548	0.0209

### Thermo gravimetric analysis

#### Thermo gravimetric analysis results

##### Thermogravimetric analysis of Diding

**Figure 2:**
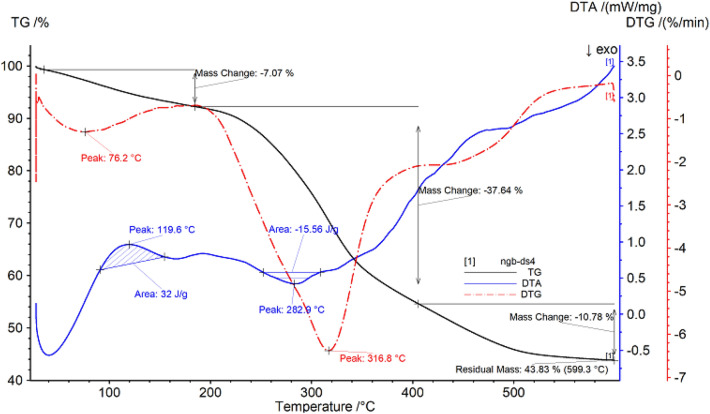
Thermogravimetric (TG %) curve, derivative thermogravimetric (DTG) curve and differential thermal analysis (DTA) curve of Diding.

The thermal gravimetric data of Diding are shown in Fig. 2 and Table 2. It can be seen from Fig. 2 that the sample begins to decompose at 34.8 °C, which may be due to the thermal desorption of the residual small molecular substances in the sample, resulting in a small amount of mass loss of the sample, with a loss rate of 7.07%; After heating for a period of time, the temperature reaches 184.1 °C and enters the second stage of decomposition. A large amount of mass loss begins to appear in the sample until 404.05 °C, and the loss rate is 37.64%; then, with the continuous increase of temperature, the sample is further decomposed, and the mass of the remaining sample is 43.83%.

**Table 2:** Thermal analysis data of Diding.

Sample	Project	Temperature range/°C	Percentage weight loss/%	The fastest weight loss temperature/°C
Diding	Peak1	34.8–184.1	7.07	76.2
	Peak2	184.1–404.5	37.64	316.8

With the increase of temperature, the DTG curve shows two peak shapes, and the inflection points of the peak shapes are 76.2 °C and 316.8 °C respectively. In addition, the DTA curve of Diding has an exothermic peak, with a peak value of 119.6 °C, a temperature range of 91.5–153.1 °C, and a peak area of 32 J/g; there is a smaller endothermic peak, with a peak value of 282.9 °C, and the temperature range is 252.8–308.6 °C, the peak area is 15.58 J/g.

##### Thermogravimetric analysis of Purslane

**Figure 3:**
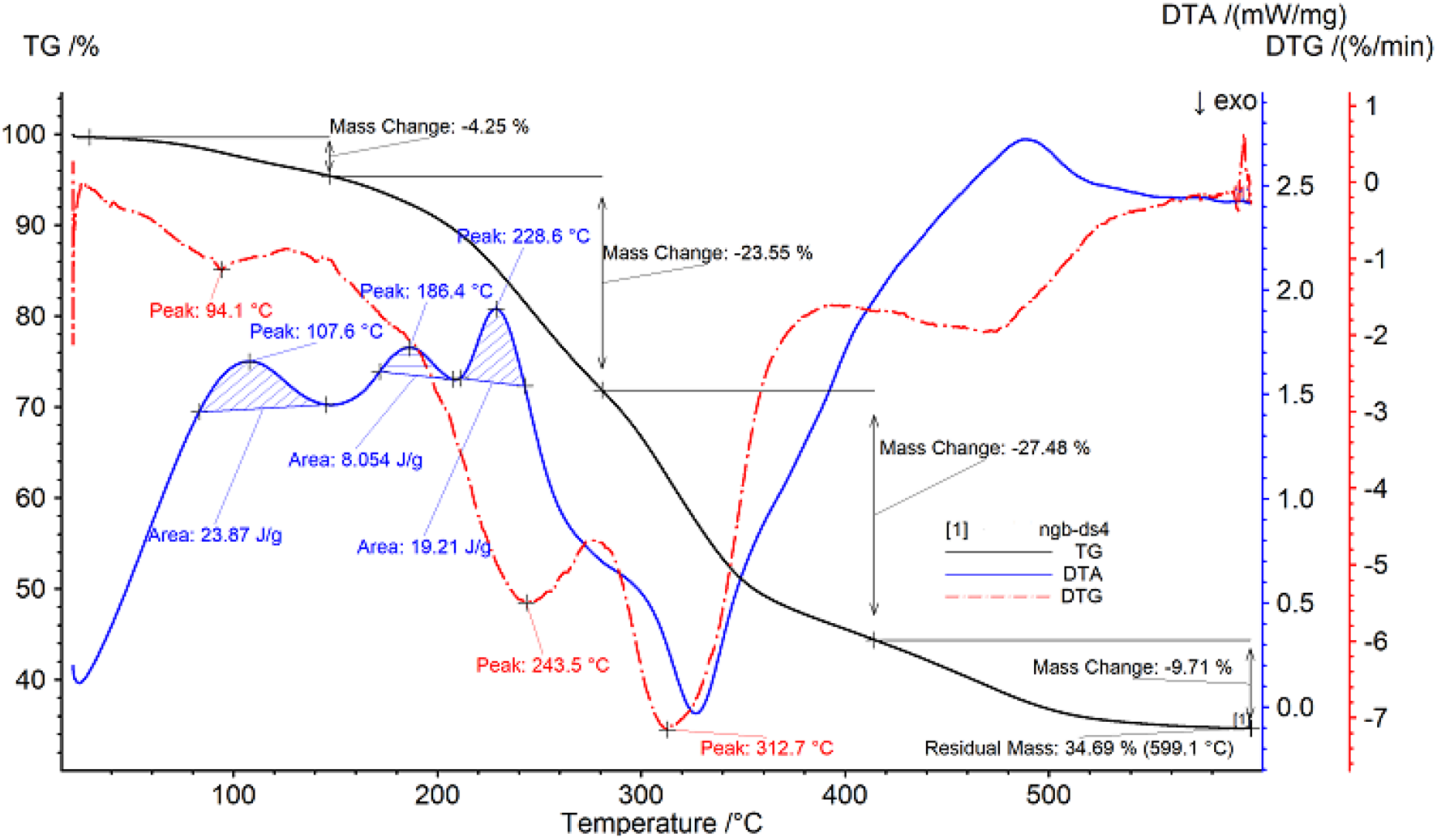
Thermogravimetric (TG %) curve, derivative thermogravimetric (DTG) curve and differential thermal analysis (DTA) curve of Purslane.

The thermal gravimetric data of Purslane are shown in Fig. 3 and Table 3. It can be seen from Fig. 3 that the sample begins to decompose at 31.3 °C, which may be due to the thermal desorption of the residual small molecular substances in the sample, resulting in a small amount of mass loss of the sample, with a loss rate of 4.25%; After heating for a period of time, the temperature reaches 146.9 °C, entering the second stage of decomposition, the sample begins to have a large mass loss until 279.7 °C, and the loss rate is 23.55%; As the temperature continues to rise, it enters the third stage of decomposition until 414.9 °C, the loss rate is 27.48%, the temperature continues to rise, the sample continues to decompose, and finally the mass of the remaining sample is 34.69%.

**Table 3:** Thermal analysis data of Purslane.

Sample	Project	Temperature range/°C	Percentage weight loss/%	The fastest weight loss temperature/°C
Purslane	Peak1	31.3–146.9	4.25	94.1
	Peak2	146.9–279.7	23.55	243.5
	Peak3	279.7–414.9	27.48	312.7

With the increase of temperature, the DTG curve shows three peaks, and the inflection points of the peaks are 94.1 °C, 243.5 °C and 312.7 °C respectively. In addition, the DTA curve of Purslane has three exothermic peaks, the first peak is 107.6 °C, the temperature range is 83.1–145.0 °C, and the peak area is 23.87 J/g; The second peak is 186.4 °C, the temperature range is 171.4–207.3 °C, and the peak area is 8.054 J/g; The third peak is 228.6 °C, the temperature range is 211.2–242.8 °C, and the peak area is 19.21 J/g.

##### Thermogravimetric analysis of straw sandal board

**Figure 4:**
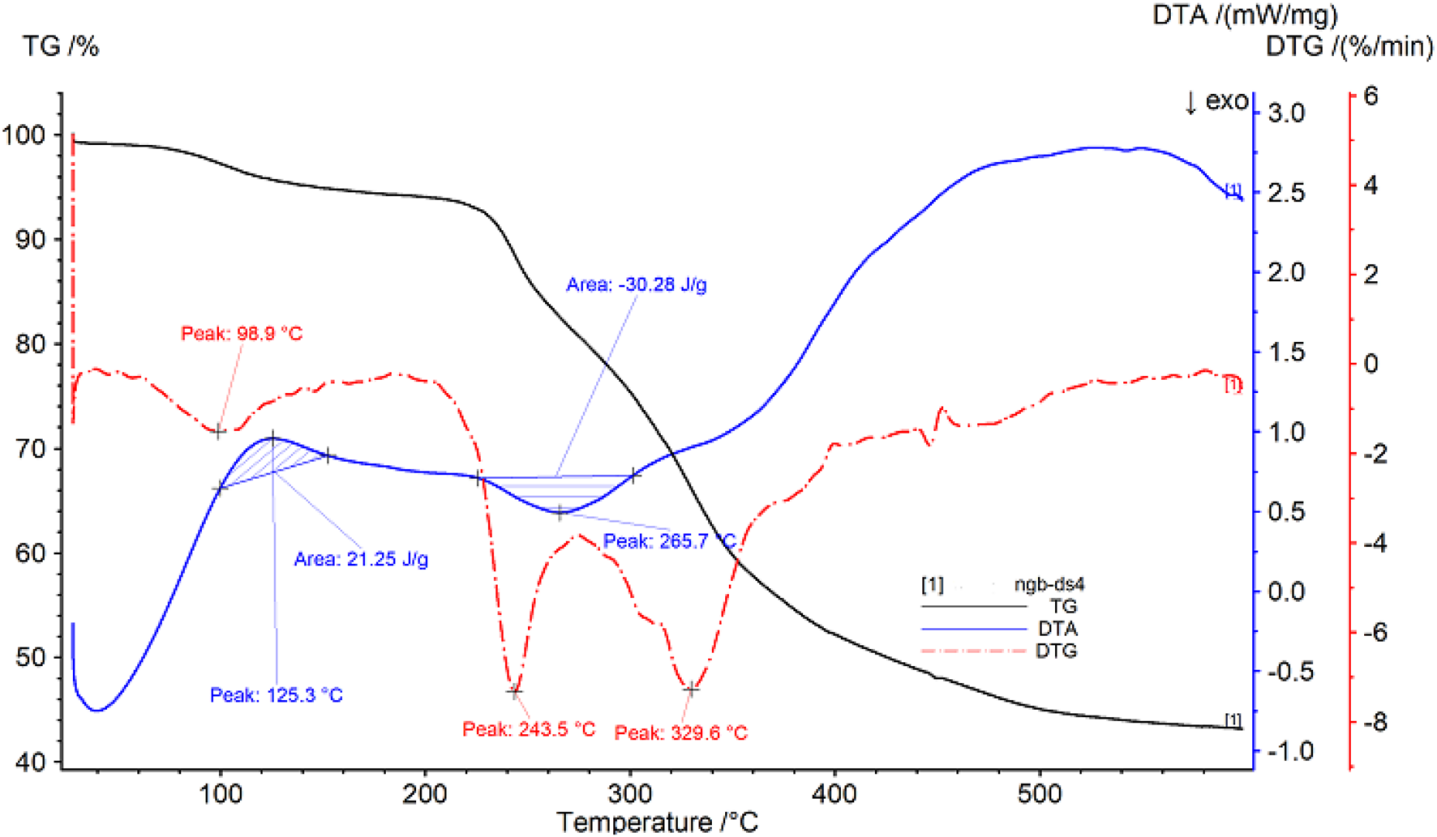
Thermogravimetric (TG %) curve, derivative thermogravimetric (DTG) curve and differential thermal analysis (DTA) curve of straw sandal board.

The thermal gravimetric data of straw sandal board are shown in Fig. 4 and Table 4. It can be seen from Fig. 4 that the sample begins to decompose at 60.8 °C. This may be due to the thermal desorption of the remaining small molecules in the sample, causing a small amount of mass loss in the sample, with a loss rate of 4.77%; after a period of time, When the temperature is increased, the temperature reaches 192.8 °C and enters the second stage of decomposition. The sample begins to show a large mass loss until 281.4 °C, and the loss rate is 14.65%; as the temperature continues to rise, it enters the third stage of decomposition until 410.6 °C, the loss rate is 28.35%, the temperature continues to rise, the sample continues to decompose, and finally the remaining sample mass is 43.18%.

**Table 4:** Thermal analysis data of straw sandal board.

Sample	Project	Temperature range/°C	Percentage weight loss/%	The fastest weight loss temperature/°C
Straw sandal board	Peak1	60.8–192.8	4.77	98.9
	Peak2	192.8–281.4	14.65	243.5
	Peak3	281.4–410.6	28.35	329.6

With the increase of temperature, the DTG curve of straw sandal board shows three peak shapes, and the inflection points of the peak shapes are 98.9 °C, 243.5 °C and 329.6 °C respectively. In addition, the DTA curve has an exothermic peak, with a peak value of 125.3 °C, a temperature range of 100.3–152.3 °C, and a peak area of 21.25 J/g; there is an endothermic peak with a peak value of 265.7 °C and a temperature range of 225.3–302.0 °C, the peak area is 30.28 J/g.

##### Thermo gravimetric analysis of June snow

**Figure 5:**
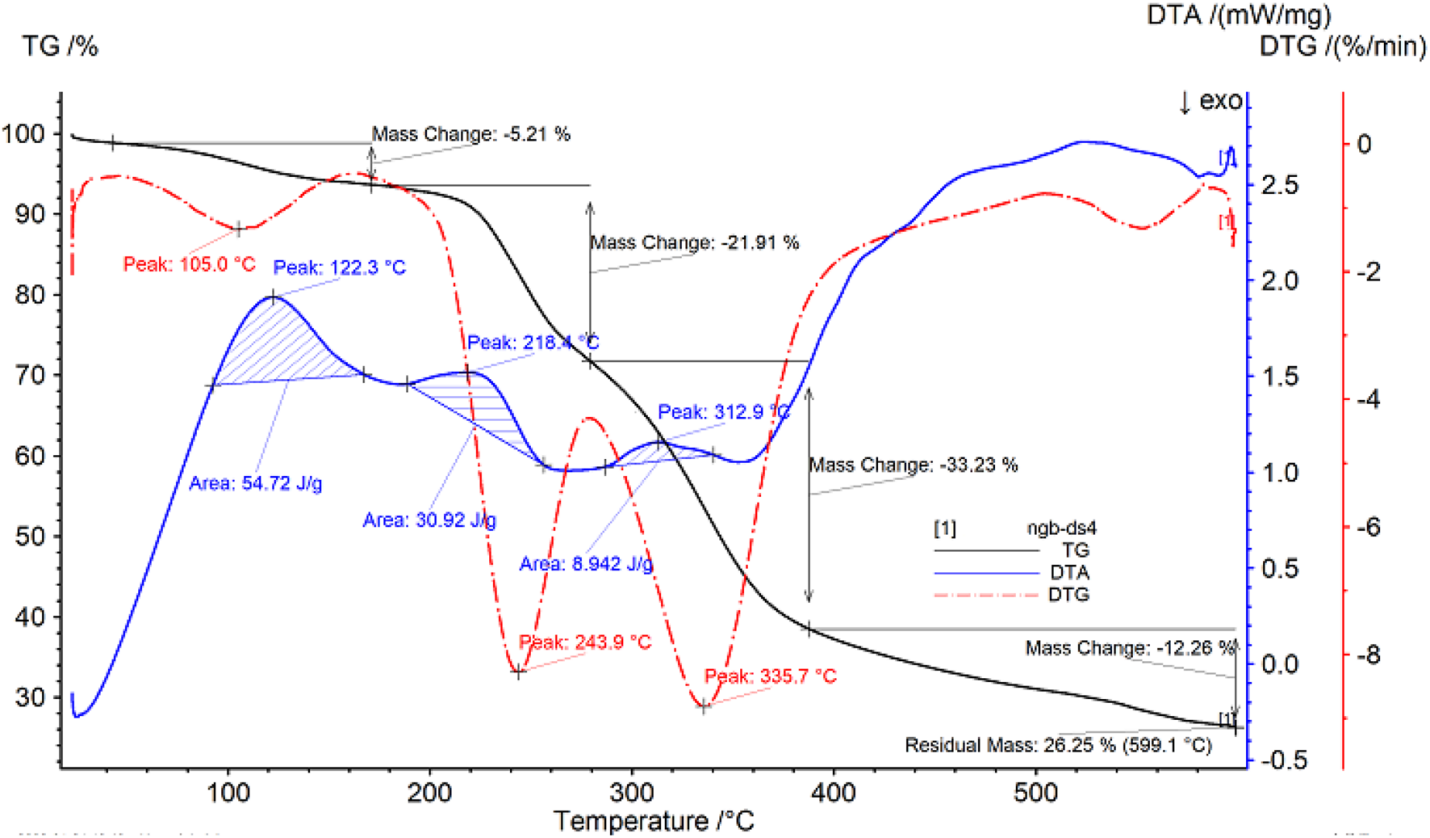
Thermogravimetric (TG %) curve, derivative thermogravimetric (DTG) curve and differential thermal analysis (DTA) curve of June snow.

The thermal gravimetric data of June snow are shown in Fig. 5, and Table 5. It can be seen from Fig. 5 that the sample begins to decompose at 42.6 °C, which may be due to the thermal desorption of the residual small molecular substances in the sample, resulting in a small amount of mass loss of the sample, with a loss rate of 5.21%; After heating for a period of time, the temperature reaches 170.5 °C and enters the second stage of decomposition until 278.7 °C, and the loss rate is 21.91%; As the temperature continues to rise and enters the third stage of decomposition, the sample begins to have a large mass loss until 387.5 °C, the loss rate is 33.23%, the temperature continues to rise, the sample continues to decompose, and finally the mass of the remaining sample is 26.25%.

**Table 5:** Thermal analysis data of June snow.

Sample	Project	Temperature range/°C	Percentage weight loss/%	The fastest weight loss temperature/°C
June snow	Peak1	42.6–170.5	5.21	105.0
	Peak2	170.5–278.7	21.91	243.9
	Peak3	287.7–387.5	33.23	335.7

With the increase of temperature, the DTG curve of June snow presents three peaks, and the inflection points of the peaks are 105.0 °C, 243.9 °C and 335 °C respectively. In addition, the DTA curve has three exothermic peaks, the first peak is 122.3 °C, the temperature range is 92.2–167.2 °C, and the peak area is 54.72 J/g; The second peak value is 218.4 °C, the temperature range is 188.3–256.2 °C, and the peak area is 30.92 J/g; The third peak is 312.9 °C, the temperature range is 286.4–340.3 °C, and the peak area is 8.942 J/g.

##### Thermo gravimetric analysis of pine vine rattan

**Figure 6:**
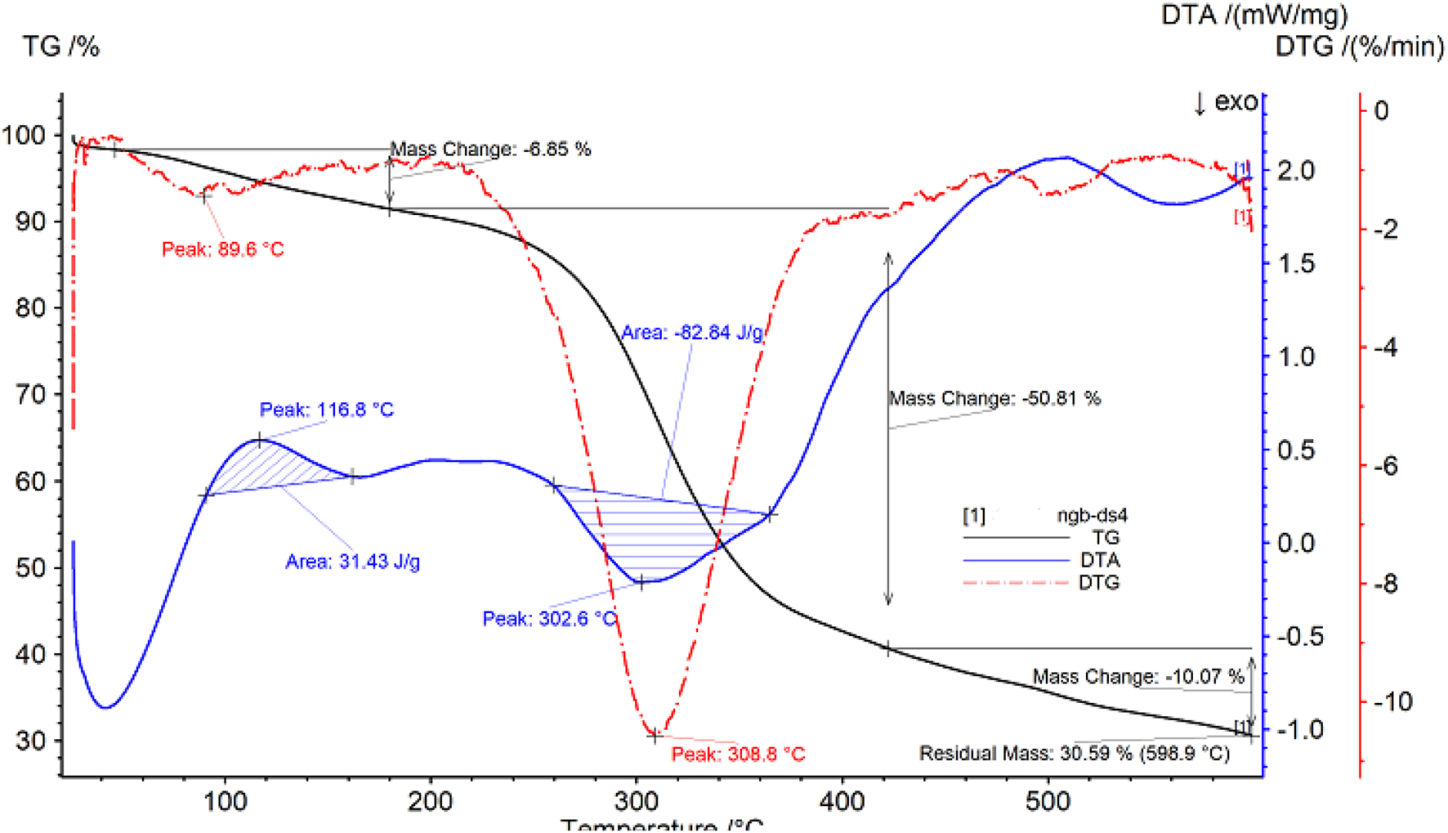
Thermo gravimetric (TG %) curve, derivative thermo gravimetric (DTG) curve and differential thermal analysis (DTA) curve of pine vine rattan.

The thermal gravimetric data of pine vine rattan are shown in Fig. 6 and Table 6. It can be seen from Fig. 6 that the sample begins to decompose at 46.9 °C. This may be due to the thermal desorption of the remaining small molecules in the sample, causing a small amount of mass loss in the sample, with a loss rate of 6.85%; after a period of heating up, the temperature reaches 180.3 °C, and enter the second stage of decomposition, the sample begins to show a large amount of mass loss, until 422.4 °C, the loss rate is 50.81%; the temperature continues to rise, the sample further decomposes, and the remaining sample mass is 30.59%.

**Table 6:** Thermal analysis data of pine vine rattan.

Sample	Project	Temperature range/°C	Percentage weight loss/%	The fastest weight loss temperature/°C
Pine vine rattan	Peak1	46.9–180.3	6.85	89.6
	Peak2	180.3–422.4	50.81	308.8

With the increase of temperature, the DTG curve of pine vine rattan shows two peak shapes, and the inflection points of the peak shapes are 89.6 °C and 308.8 °C respectively. In addition, the DTA curve has an exothermic peak, with a peak value of 116.8 °C, a temperature range of 90.9–161.7 °C, and a peak area of 31.43 J/g; there is a larger endothermic peak with the peak value of 302.6 °C and a temperature range of 259.8–364.5 °C, and the peak area is 82.84 J/g.

### Combustion stability analysis of 5 kinds of genuine medicinal materials

The thermogravimetric parameters, including index X_1_ the first stage weight loss percentage, index X_2_ the fastest temperature in the first stage of weight loss, index X_3_ the weight loss percentage in the second stage, index X_4_ the fastest temperature in the second stage of weight loss, index X_5_ the percentage of weight loss in the third stage, index X_6_ the remaining mass percentage, index X_7_ the peak area of the first stage and index X_8_ the second stage peak area, were used to build combustion stability of genuine medicinal materials through gray pattern recognition. The thermal gravimetric parameter data of 5 kinds of genuine medicinal materials were shown in Table S1. Table S1 is in Supplementary Materials. Based on the thermogravimetric parameter data of five genuine medicinal materials, a multi-index evaluation system for combustion stability was established. Thermo gravimetry is to study the combustion characteristic index of genuine medicinal particles at different heating rates by thermo gravimetry analyzer to judge the combustion stability of genuine medicinal materials. According to the method of grey pattern recognition^56^, this subject calculates the correlation coefficient between each scheme and the ideal scheme composed of the best indicators, obtains the correlation degree from the correlation coefficient, and then sorts and analyzes it according to the correlation degree to draw a conclusion. The greater the correlation degree Z is, the better the sample effect is. Finally, compare all the Z values to draw the evaluation conclusion. Calculated by EXCEL, the Z values of 5 kinds of genuine medicinal materials, including Diding, Purslane, straw sandal board, June snow and pine vine rattan, are 0.7199, 0.7050, 0.7373, 0.8014 and 0.7749 respectively. From the analysis of thermogravimetric analysis results and thermogravimetric combustion stability, the order of combustion stability of 5 kinds of genuine medicinal materials was June snow > pine Vine rattan > straw sandal board > Diding > Portulaca oleracea.

### Determination of fat, calcium, ash content

The determination results of fat, calcium content, ash content of 5 kinds of genuine medicinal materials were shown in Table 7. Table 7 showed, the order of fat content (%) of 5 kinds of genuine medicinal materials, including Diding, Purslane, straw sandal board, June snow, pine vine rattan, was straw sandal board > Diding > pine vine rattan > June snow > Purslane, the order of calcium content (%) was pine vine rattan > June snow > Purslane > straw sandal board > Diding, the order of ash content was June snow > Purslane > straw sandal board > pine vine rattan > Diding. The energy value of genuine medicinal materials can also be reflected by the combustion heat, fat content to a certain extent. The contents of ash, fat, and calcium are regarded as important physical data to measure the quality of genuine medicinal materials. The quality of genuine medicinal materials is evaluated from the aspect of energy, which provides a strong scientific basis for the classification of genuine medicinal materials.

**Table 7:** Determination results of fat, calcium, ash content in 5 kinds of genuine medicinal materials (n = 3, CV% < 2.0%).

Sample	Fat content/%	Calcium content/%	Ash/%
Diding	0.8932	0.36	7.1864
Purslane	0.2984	0.61	20.1744
Straw sandal board	1.2568	0.49	19.8100
June snow	0.3099	0.86	21.3675
Pine vine rattan	0.3697	2.66	9.5545

### Determination of trace elements

#### Determination results of trace elements

A method for the determination of 12 trace elements, including Mn, Mg, Fe, Co, Zn, Cu, Ni, Se, Sn, As, Li and Mo in 5 genuine medicinal materials, was established by inductively coupled plasma mass spectrometry (ICP-MS) based on microwave digestion^57–61^. Average value and standard deviation of 12 trace elements in 5 kinds of genuine medicinal materials, including Diding, Purslane, straw sandal board, June snow, pine vine rattan, were shown in Table 8.

**Table 8:** Average values ± standard deviation of 12 trace elements in 5 kinds of genuine medicinal materials (μg/g, n = 6).

Sample	Diding	Purslane	Straw sandal board	June snow	Pine vine rattan
As	0.0913 ± 0.00058	0.0897 ± 0.00231	0.0637 ± 0.00416	0.9567 ± 0.03055	0.1983 ± 0.25260
Co	0.1233 ± 0.00577	0.1300 ± 0.14000	0.1267 ± 0.00577	0.1647 ± 0.00208	0.1117 ± 0.08431
Cu	18.3400 ± 0.09644	15.4933 ± 0.09815	23.9367 ± 0.14364	34.4500 ± 0.07000	17.7300 ± 0.23065
Fe	1878.4400 ± 11.48402	1053.2267 ± 4.77611	3047.7133 ± 21.37930	4137.2567 ± 8.98656	59.5800 ± 0.26230
Li	2.0900 ± 0.01000	1.3500 ± 0.01000	1.0800 ± 0.01000	0.5567 ± 0.00577	0.0100 ± 0.00000
Mg	2561.1800 ± 1.69779	3401.0000 ± 4.19858	1790.4100 ± 0.20952	1092.7800 ± 0.56956	879.9300 ± 1.20200
Mn	88.9867 ± 0.28290	35.6267 ± 0.27025	155.9667 ± 0.40079	210.0433 ± 0.42712	43.2000 ± 0.34828
Mo	0.1047 ± 0.00252	0.1897 ± 0.00208	0.1390 ± 0.00700	0.0483 ± 0.00379	0.0433 ± 0.00577
Ni	2.8633 ± 0.37207	2.0333 ± 0.28537	3.2000 ± 0.14731	6.8733 ± 0.23459	1.3167 ± 0.18148
Se	0.0743 ± 0.00153	0.0447 ± 0.00153	0.0803 ± 0.00058	0.1070 ± 0.00100	0.0467 ± 0.00208
Sn	0.1147 ± 0.01258	0.1167 ± 0.00577	0.0860 ± 0.00361	0.0587 ± 0.00451	0.1520 ± 0.01136
Zn	42.4300 ± 0.14107	21.6600 ± 0.52163	117.3667 ± 0.57003	28.9767 ± 0.38682	18.8767 ± 0.12662

### Grey factor analysis of 12 trace elements in 5 kinds of genuine medicinal materials^62–66^

Through grey factor analysis, the characteristic roots of grey factor correlation coefficient matrix and variance contribution rate of trace elements, including Mn, Mg, Fe, Co, Zn, Cu, Ni, Se, Sn, as, Li and Mo, are obtained, as shown in Table 9. According to Table 9, the cumulative contribution rate of the main factors of the first three grey factors reaches 93.403%, and the eigenvalues of the main factors of the first three grey correlation coefficient factors (λ > 1) are larger, that is, the main factors of the first three grey factors contribute the most to the explanatory variables. It is most appropriate to extract the main factors of the first three grey correlation coefficient factors, which represents 93.403% of the information of 12 trace elements in the five genuine medicinal materials in Guangxi, China.

**Table 9:** Grey factor coefficient characteristic root and variance contribution rate of 12 trace elements in 5 kinds of genuine medicinal materials.

Grey principal factor	Characteristic root	Contribution rate%	Cumulative contribution rate %
1	7.752	64.599	64.599
2	2.095	17.457	82.055
3	1.362	11.348	93.403
…	…	…	…

The gray correlation coefficient factor load matrix after rotation is shown in Table 10. It can be seen from Table 10 that the first main factor F_1_ of the gray correlation coefficient mainly contains the original variables As, Co, Cu, Fe, Mn, Ni, Se trace element information necessary for the human body. The second main factor F_2_ of the gray correlation coefficient mainly contains the information of the original variables Li, Mg and Mo. The third main factor F of the gray correlation coefficient mainly contains the information of the original variables Zn and Sn.

**Table 10:** The rotated grey correlation coefficient factor loading matrix of 12 trace elements in 5 kinds of genuine medicinal materials.

Sample	Grey correlation factor 1	Grey correlation factor 2	Grey correlation factor 3
As	0.901	− 0.261	− 0.336
Co	0.964	0.049	− 0.225
Cu	0.959	− 0.278	− 0.004
Fe	0.981	− 0.062	0.183
Li	− 0.147	0.676	0.043
Mg	− 0.31	0.925	− 0.179
Mn	0.961	− 0.197	0.194
Mo	− 0.285	0.848	0.136
Ni	0.976	− 0.141	− 0.167
Se	0.961	− 0.14	0.118
Sn	− 0.895	− 0.324	− 0.306
Zn	0.073	0.035	0.994

The gray correlation coefficient factor score and the comprehensive gray correlation coefficient factor score are shown in Table 11. As can be seen from Table 11, the order of the content of 12 trace elements in 5 kinds of genuine medicinal materials, including Diding, Purslane, straw sandal board, June snow, pine vine rattan, is June snow > straw sandal board > Diding > Purslane > pine vine rattan. In terms of trace element content, June snow contains the highest trace elements in all samples.

**Table 11:** Grey correlation coefficient factors and comprehensive factor scores of 12 trace elements in 5 kinds of genuine medicinal materials.

Sample	F_1_	F_2_	F_3_	F	Ranking
Diding	− 0.26823	0.54251	− 0.14727	− 0.102	3
Purslane	− 0.47017	1.25832	− 0.60818	− 0.16388	4
Straw sandal board	0.0857	− 0.0013	1.75999	0.27286	2
June snow	1.6467	− 0.39818	− 0.55849	0.99656	1
Pine vine rattan	− 0.994	− 1.40135	− 0.44606	− 1.00354	5

## Discussion

### Construction of multi-index comprehensive evaluation system for 5 kinds of genuine medicinal materials

#### Construction of multi-index analysis and comprehensive evaluation system by entropy analysis

According to the combustion heat, thermogravimetric parameters, fat content, calcium content, trace element content and ash content, the multi-index comprehensive evaluation systems of five kinds of genuine medicinal materials including Diding, Purslane, straw sandal board, June snow, pine vine rattan were established by entropy method^67–72^.

According to the characteristics of entropy, this paper judges the randomness and disorder degree of an event by calculating the entropy value, and judges the dispersion degree of an index by using the entropy value^73–76^. The greater the dispersion degree of the index is, the greater the influence (weight) of the index on the comprehensive evaluation is, and the smaller the entropy value is. Using entropy method, 5 kinds of genuine medicinal materials were weighted to calculate the comprehensive score S.

1. Standardized treatment,${\text{y}}^{\prime}_{{{\text{ij}}}} { = }\frac{{x_{{{\text{ij}}}} - x_{j\min } }}{{x_{j\max } - x_{j\min } }}$, where $y^{\prime}_{ij}$( i = 1,2,…, n ; j = 1,2,…, m ) is the j index value of the i sample after dimensionless treatment, the original data of the j index of the i sample is the maximum value of the j index and the minimum value of the j index.
2. Calculation of the proportion of sample i under indicator j $${\text{P}}_{ij}$$$$(0 \le P_{ij} \le 1)$$$$P_{ij} = \frac{{y^{\prime}_{ij} }}{{\sum\nolimits_{i = 1}^{{\text{n}}} {y^{\prime}_{ij} } }}.$$
3. Information entropy value e and information utility value d, information entropy value of item j is $e_{j} = - \frac{1}{\ln m}\sum\nolimits_{i = 1}^{{\text{n}}} {P_{ij} } {\text{lnP}}_{ij}$ Information utility value $d_{j} = 1 - e_{j} .$
4. Weight of evaluation indicators. The greater the information utility value is, indicating that the more important the indicators, the greater the importance of evaluation is. Finally, the weight of the j index is $W_{j} = \frac{{d_{j} }}{{\sum\nolimits_{j = 1}^{{\text{m}}} {d_{j} } }}$
5. Comprehensive evaluation $$S = \sum\nolimits_{j = 1}^{m} {(W_{j} P_{ij} } ).$$

The weighted summation formula is used to calculate the evaluation value of the sample. The larger the comprehensive score S is, the better the sample effect is. Finally, compare all S values, that is, draw the evaluation conclusion. Using EXCEL calculation, the S values of Diding, Purslane, straw sandal board, June snow, pine vine rattan were 0.3764, 0.2777, 0.4876, 0.5744 and 0.2688 respectively.

According to combustion heat, combustibility (combustion stability of genuine medicinal materials), fat, calcium, ash, trace element content, the comprehensive evaluation results of multi-index analysis constructed by entropy analysis showed that the comprehensive evaluation multi-index order of 5 genuine medicinal materials, including Diding, Purslane, straw sandal board, June snow and pine vine rattan, was June snow > straw sandal board > Diding > Purslane > pine vine rattan. Therefore, the comprehensive evaluation results of the quality of genuine medicinal materials selected in this study were June snow the best, followed by straw sandal board.

#### Construction of multi-index analysis and comprehensive evaluation system by grey correlation coefficient cluster analysis

**Figure 7:**
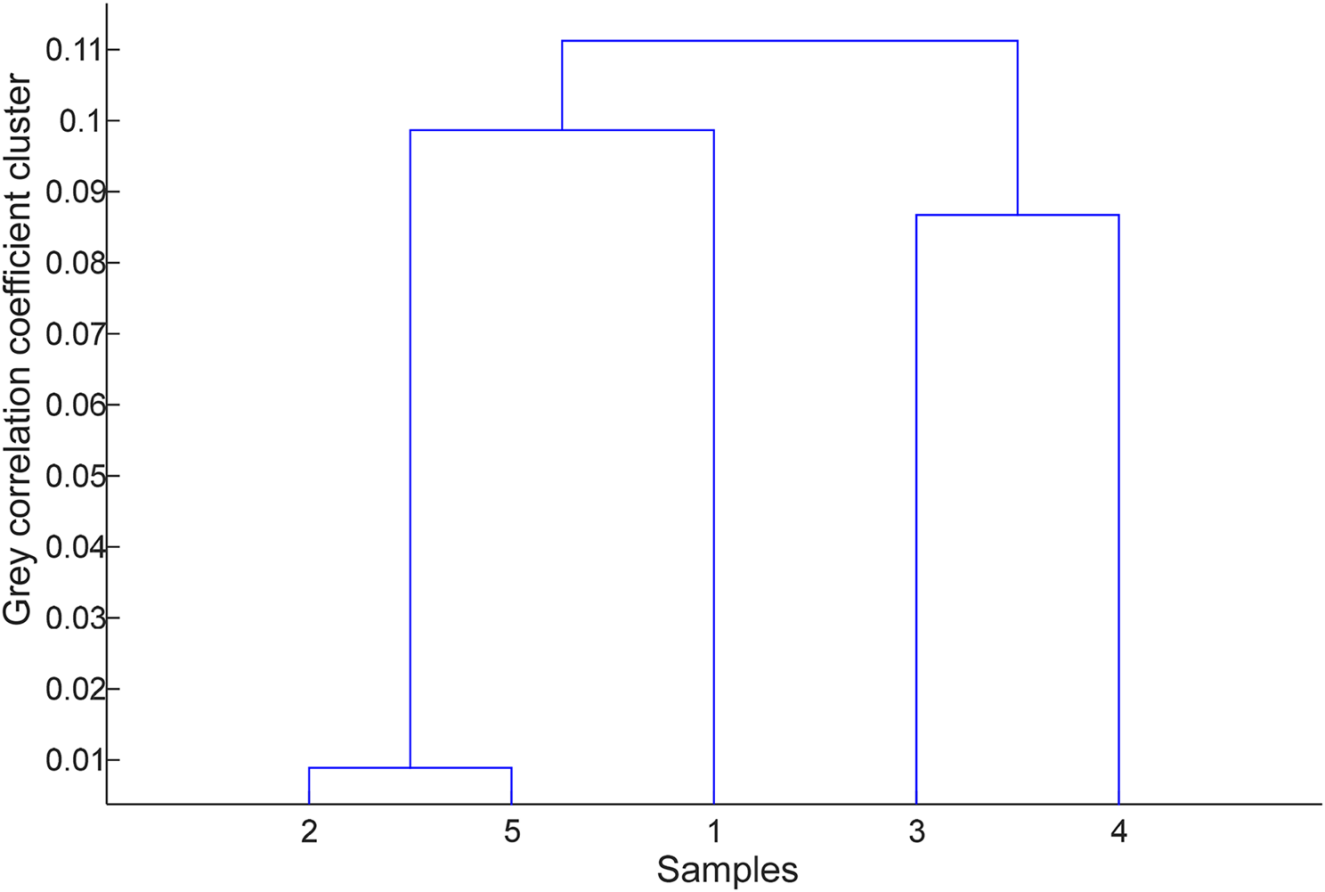
Tree diagram of grey correlation coefficient cluster analysis of multiple indexes of 5 kinds of genuine medicinal materials.

The gray correlation coefficient cluster analysis is based on the many properties of the sample, and the cluster analysis diagram is obtained from the correlation coefficient^77–80^. According to the literature^81,82^, the classification is carried out according to the degree of affinity of the nature of the sample. All cases are classified into different classes, making the same class Individuals in different classes have greater similarities, and individuals in different classes have greater differences. The multi-index comprehensive cluster analysis system of combustion heat, combustibility (combustion stability of genuine medicinal materials), fat content, calcium, ash and trace element content of 5 genuine medicinal materials in Guangxi was established. The gray correlation coefficient cluster analysis tree diagram was shown in Fig. 7. As can be seen from Fig. 7, 5 kinds of genuine medicinal materials from different producing areas, namely Diding, Purslane, straw sandal board, June snow and pine vine rattan, were divided into three categories according to the results of grey correlation coefficient cluster analysis. Straw sandal board, June snow were a class, Diding for one class, and Purslane and pine vine rattan for a class. Through the grey correlation coefficient cluster analysis, we found the similarity degree and genetic relationship between the properties of genuine medicinal materials from different origins, which can help better study the classification of genuine medicinal materials.

## Conclusion

Thermogravimetric parameters were applied to the evaluation of the combustion stability of genuine medicinal materials through gray pattern recognition, which provided a strong scientific basis for the evaluation and research of the combustion stability of genuine medicinal materials by thermogravimetric analysis.

A method for the determination of 12 trace elements, including Mn, Mg, Fe, Co, Zn, Cu, Ni, Se, Sn, As, Li and Mo in 5 genuine medicinal materials, was established by inductively coupled plasma mass spectrometry (ICP-MS) based on microwave digestion, and the data were comprehensively analyzed by the grey factor analysis method. From the content of trace elements, the trace elements contained in Junxue were the highest in all samples. These studies are for scientific research on trace elements and biological activities in medicinal materials, for the rational development of the quality control of Chinese medicinal materials in the standardized planting base of Chinese medicinal materials in Guangxi, and to provide reference for the medical and health care of Chinese medicinal materials.

In this paper, according to combustion heat, differential thermal-thermogravimetric analysis, fat content, calcium and ash content, trace element content data of genuine medicinal materials, systematic multi-index comprehensive evaluation systems were constructed through gray pattern recognition, gray factor analysis, entropy method and gray correlation coefficient cluster analysis. As long as the various parameters like combustion heat, thermogravimetric parameters, and fat content, calcium content, trace element content, ash content, etc. are measured, systematic multi-index comprehensive evaluation systems are constructed through gray pattern recognition, gray factor analysis, entropy method and gray correlation coefficient cluster analysis, and then can be utilized for the identification of genuine materials. This research has important theoretical and practical significance for the multi-index measurement and comprehensive evaluation of genuine medicinal materials, and can provide scientific basis and research significance for the research of multi-index quality control of genuine medicinal material.

The multi-index comprehensive evaluation system established in this study provides a new idea for the quantitative control of the quality of genuine medicinal materials, and provides a powerful way for the large-scale development and classification research of genuine medicinal materials and provides basic support for the selection of raw materials of genuine medicinal materials and the application of quantitative control mode of multi-index ingredients to the quality control of genuine medicinal materials.

## Supplementary Information

Supplementary Information.
