# Supplementary material for: Entropy analysis and grey cluster analysis of multiple indexes of 5 kinds of genuine medicinal materials
Source: Sci Rep. 2022 Apr 22;12:6618. doi: 10.1038/s41598-022-10509-0 (PMC9033816; doi:10.1038/s41598-022-10509-0)
Supplement: Supplementary file 1 — Supplementary Information. [file 41598_2022_10509_MOESM1_ESM.docx]

Supplementary data

Fig. S1 Diding


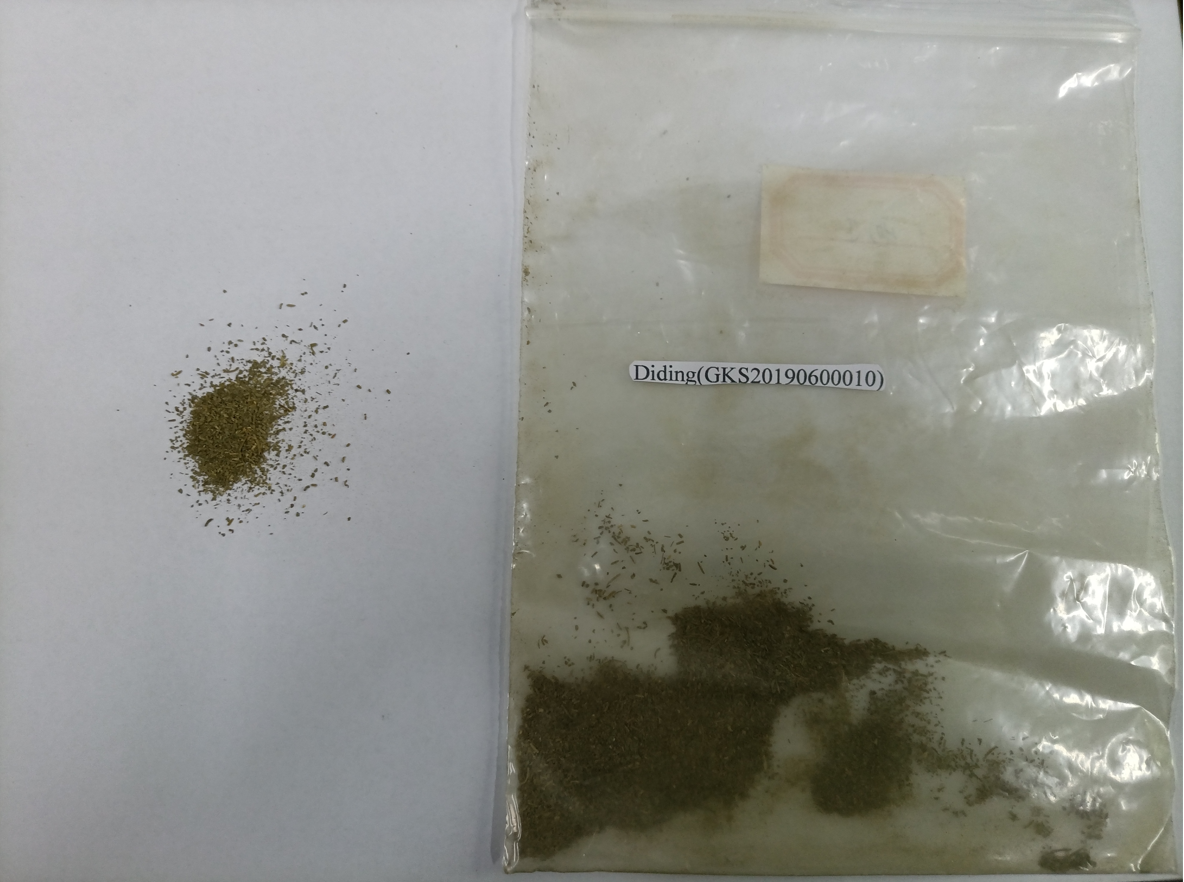


Fig. S2 Purslane


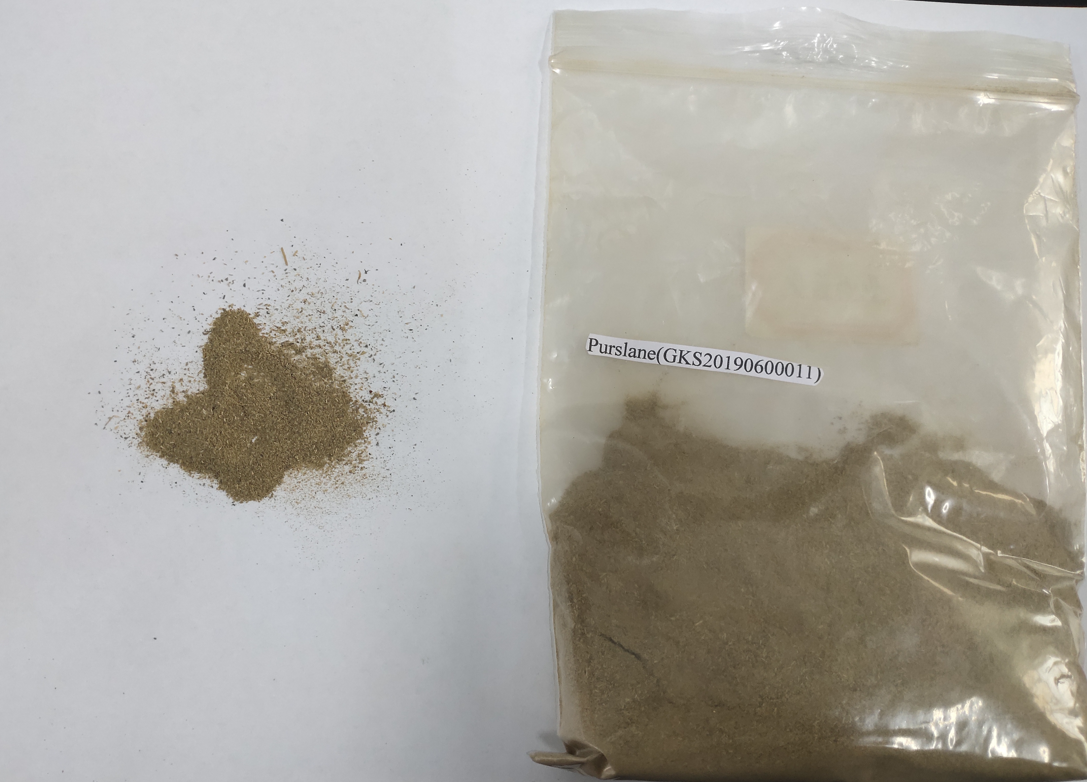


Fig. S3 Straw sandal board


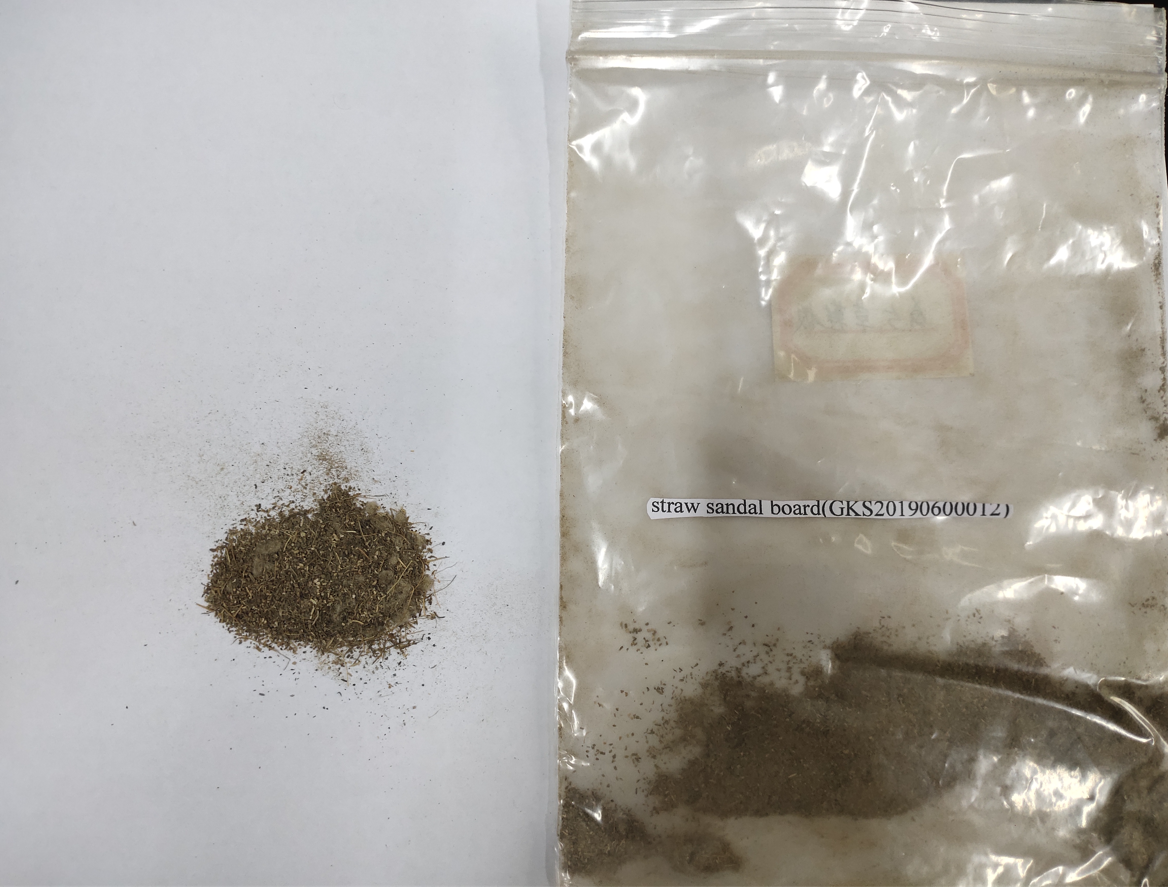


Fig. S4 June snow


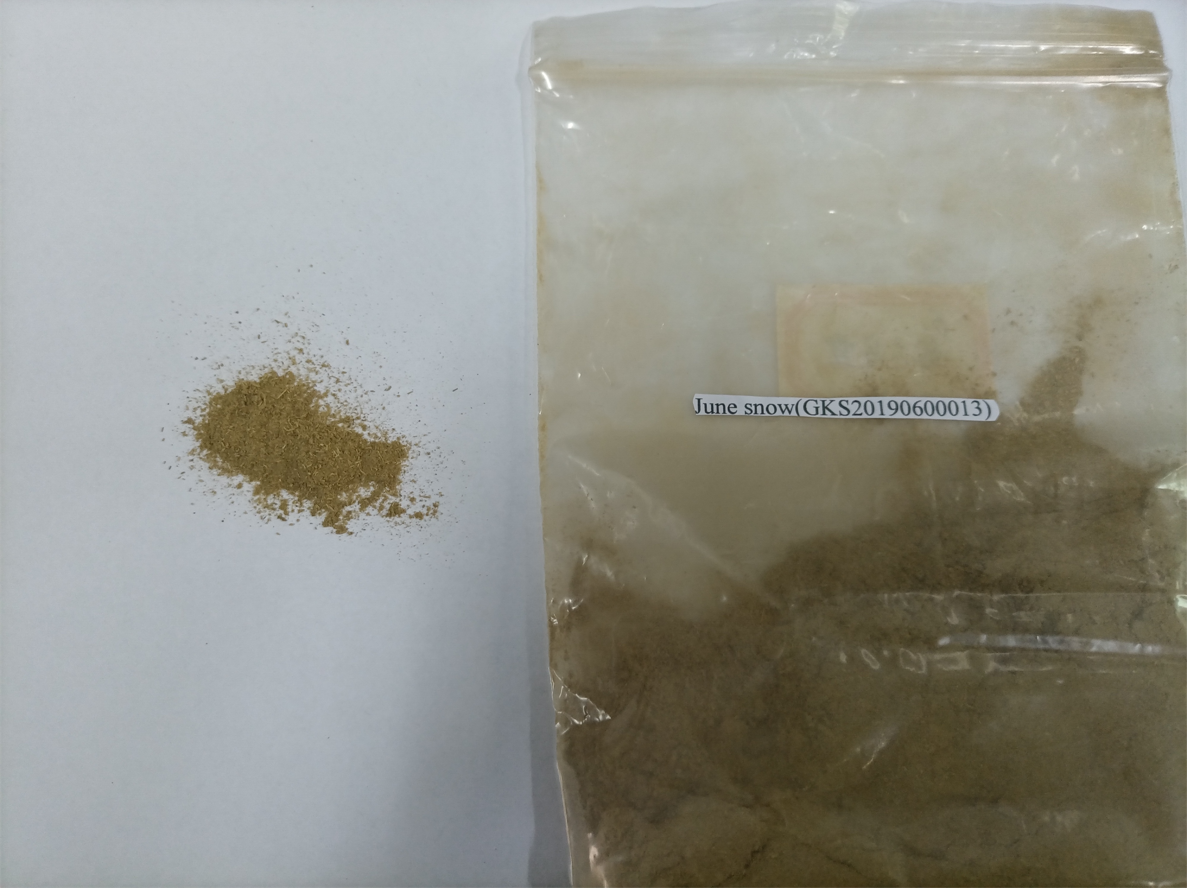


Fig. S5 Pine vine rattan


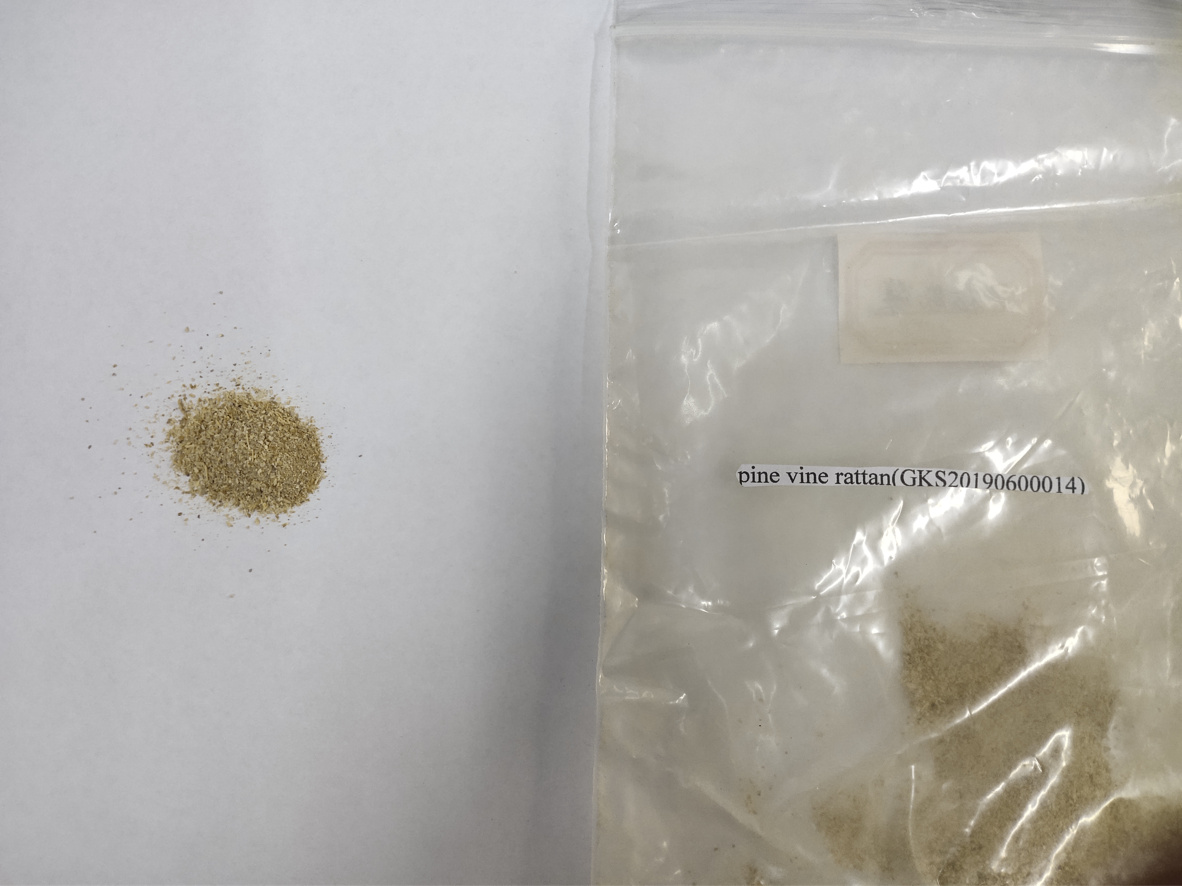


Table S1 The thermal gravimetric parameter data of 5 kinds of genuine medicinal materials

| Sample | Diding | Purslane | straw sandal board | June snow | pine vine rattan |
| --- | --- | --- | --- | --- | --- |
| Sample mass /mg | 5.4 | 6.7 | 5.2 | 5.2 | 4.1 |
| Crucible mass /mg | 170.2 | 188.7 | 173.5 | 187.2 | 177.3 |
| IndicatorX_1_/% | 7.07 | 4.25 | 4.77 | 5.21 | 6.85 |
| IndicatorX_2_/℃ | 76.2 | 94.1 | 98.9 | 105 | 89.6 |
| IndicatorX_3_/% | 37.64 | 23.55 | 14.65 | 21.91 | 50.81 |
| IndicatorX_4_/℃ | 316.8 | 245.5 | 243.5 | 243.9 | 308.8 |
| IndicatorX_5_/% | 10.78 | 27.48 | 28.35 | 33.23 | 10.07 |
| IndicatorX_6_/% | 43.83 | 34.6 | 43.1 | 26.25 | 30.59 |
| IndicatorX_7_/J/g | 32 | 23.87 | 21.25 | 54.72 | 31.43 |
| IndicatorX_8_/J/g | 15.56 | 8.054 | 30.28 | 30.92 | 82.84 |
